# The role of macrophages in radiation-induced lung injury: from pathological mechanisms to therapeutic targets

**DOI:** 10.3389/fimmu.2026.1863541

**Published:** 2026-07-22

**Authors:** Xiaochi Ma, Jie Lu, Tao Zhong, Xin Wang, Xiao Liu, Wen Huo, Xiaozheng Chen

**Affiliations:** 1 Department of Pulmonary and Critical Care Medicine, Qilu Hospital of Shandong University, Cheeloo College of Medicine, Shandong University, Jinan, Shandong, China; 2 Department of Critical Care Medicine, Shandong Provincial Hospital Affiliated to Shandong First Medical University, Jinan, Shandong, China; 3 Institute of Immunology and Molecular Medicine, Key Laboratory of Cell and Biomedical Technology of Shandong Province, College of Basic Medicine, Jining Medical University, Jining, Shandong, China; 4 Department of Radiation Oncology, Shandong First Medical University and Shandong Academy of Medical Sciences, Shandong Cancer Hospital and Institute, Jinan, Shandong, China; 5 Department of Radiation Oncology, Affiliated Tumor Hospital of Xinjiang Medical University, Urumqi, Xinjiang, China; 6 Department of Radiation Oncology, Qilu Hospital of Shandong University, Cheeloo College of Medicine, Shandong University, Jinan, Shandong, China

**Keywords:** macrophages, phenotypic transformation, radiation-induced lung injury, radiotherapy, reprogramming, therapeutic strategies

## Abstract

Radiation-induced lung injury (RILI) is a major dose-limiting toxicity of chest radiotherapy (RT), and its occurrence and development are closely related to the complex role of macrophages. RT significantly modulates the recruitment and functional status of macrophages. In the early stage, macrophages predominantly exhibit a pro-inflammatory M1 phenotype, releasing a large amount of inflammatory factors and exacerbating lung tissue damage. In the late stage, they shift to a pro-fibrotic M2 phenotype, promoting fibroblast activation, extracellular matrix accumulation, and epithelial-mesenchymal transition, thereby facilitating the progression of pulmonary fibrosis. In addition, the metabolic reprogramming induced by RT significantly affects the function of macrophages, manifested by imbalances in glucose, lipid, and amino acid metabolism and mitochondrial dysfunction, further reinforcing phenotypic polarization and functional heterogeneity. This complexity offers diversified strategies for the treatment of RILI by targeting macrophages, including phenotypic regulation, metabolic intervention, delivery systems and cell engineering development, and combination therapy, demonstrating new potential for mitigating RILI. However, the widespread use of immune checkpoint inhibitors has exacerbated the difficulty in preventing and treating RILI, necessitating urgent exploration of effective and safe intervention measures.

## 1 Introduction

Currently, thoracic tumors have high incidence and mortality rates worldwide (1). Radiotherapy (RT) is one of the most crucial treatment methods for thoracic malignancies. While radiation can effectively kill tumor cells and improve the prognosis of patients, it inevitably damages normal thoracic tissues and organs. Radiation-induced lung injury (RILI), defined as a spectrum of pathological changes in normal lung parenchyma after exposure to radiation, is one of the most serious complications, with an incidence of up to 31.4% (2). Clinically, it is divided into two stages: the acute stage (1–6 months after RT), known as radiation pneumonitis (RP), and the chronic stage (6–24 months after RT), characterized by radiation-induced lung fibrosis (RILF). Current RILI treatment mainly relies on long-term glucocorticoids, immunosuppressants and antifibrotic agents. Long-term glucocorticoid therapy triggers osteoporosis, hypertension and sodium-water retention, thus limiting its use in RILI prevention and management. Immunosuppressants induce life-threatening complications such as severe infections, bone marrow suppression and organ toxicities. Antifibrotic drugs pirfenidone and nintedanib may benefit RILI but lack sufficient evidence (3). Therefore, clarifying RILI’s pathophysiological mechanisms and identifying new therapeutic targets are clinically valuable.

RILI involves complex interactions among innate and adaptive immune cells. T-cell responses are stage- and subset-dependent: thoracic irradiation induces local and systemic CD4^+^FoxP3^+^ Treg accumulation, which may restrain excessive acute inflammation (4), whereas persistent Treg activation can promote RIPF through TGF-β1 secretion, EMT activation, and profibrotic signaling (5). The Th1/Th17 balance further influences the inflammatory-to-fibrotic transition (6). In contrast, the direct causal role of B cells in RILI remains less defined, although Breg/B10 cells may link immune suppression to fibrotic remodeling (7). Although adaptive immune cells contribute to the immune complexity of RILI, macrophages are arguably the most pivotal immune cells in this cascade. As highly plastic innate immune sentinels, macrophages act as the central orchestrators of the lung microenvironment. They serve as the critical bridge between radiation-induced tissue damage and the adaptive immune response, coupling the acute inflammatory phase with the chronic fibrotic phase through dynamic phenotypic reprogramming. For instance, M1 macrophages exert pro-inflammatory and anti-fibrotic effects, while M2 macrophages exhibit anti-inflammatory and pro-fibrotic effects (8). This article systematically elucidates the mechanisms by which RT induces macrophage reprogramming, reviews how signals triggered by RT recruit and activate macrophages, how time-dependent signal transduction, epigenetic and metabolic programs drive polarization and functional heterogeneity and how these insights reveal macrophage-centric therapeutic targets and rational combinations. This review aims to provide new ideas for the development of effective macrophage-targeted therapeutic strategies, to deepen the understanding of the molecular mechanisms and pathophysiology of RILI, and to facilitate the translation of basic research into clinical applications.

## 2 Radiation-induced macrophage reprogramming in the immune microenvironment

### 2.1 Radiotherapy promotes the recruitment and activation of macrophages

After RT, extensive macrophage infiltration and accumulation in lung tissue are consistently observed in multiple animal models and clinical histopathological studies. The proportion of macrophages in bronchoalveolar lavage fluid increases markedly post-irradiation, making them one of the dominant infiltrating immune cell populations (9). Quantitative analyses in mouse models demonstrate a strong positive correlation between pulmonary macrophage numbers and the severity of RILI (10).

The mechanisms by which RT induces macrophage recruitment are complex and multifaceted, primarily involving the following pathways:

#### 2.1.1 Direct radiation injury can induce the release of damage-associated molecular patterns

**Figure 1:**
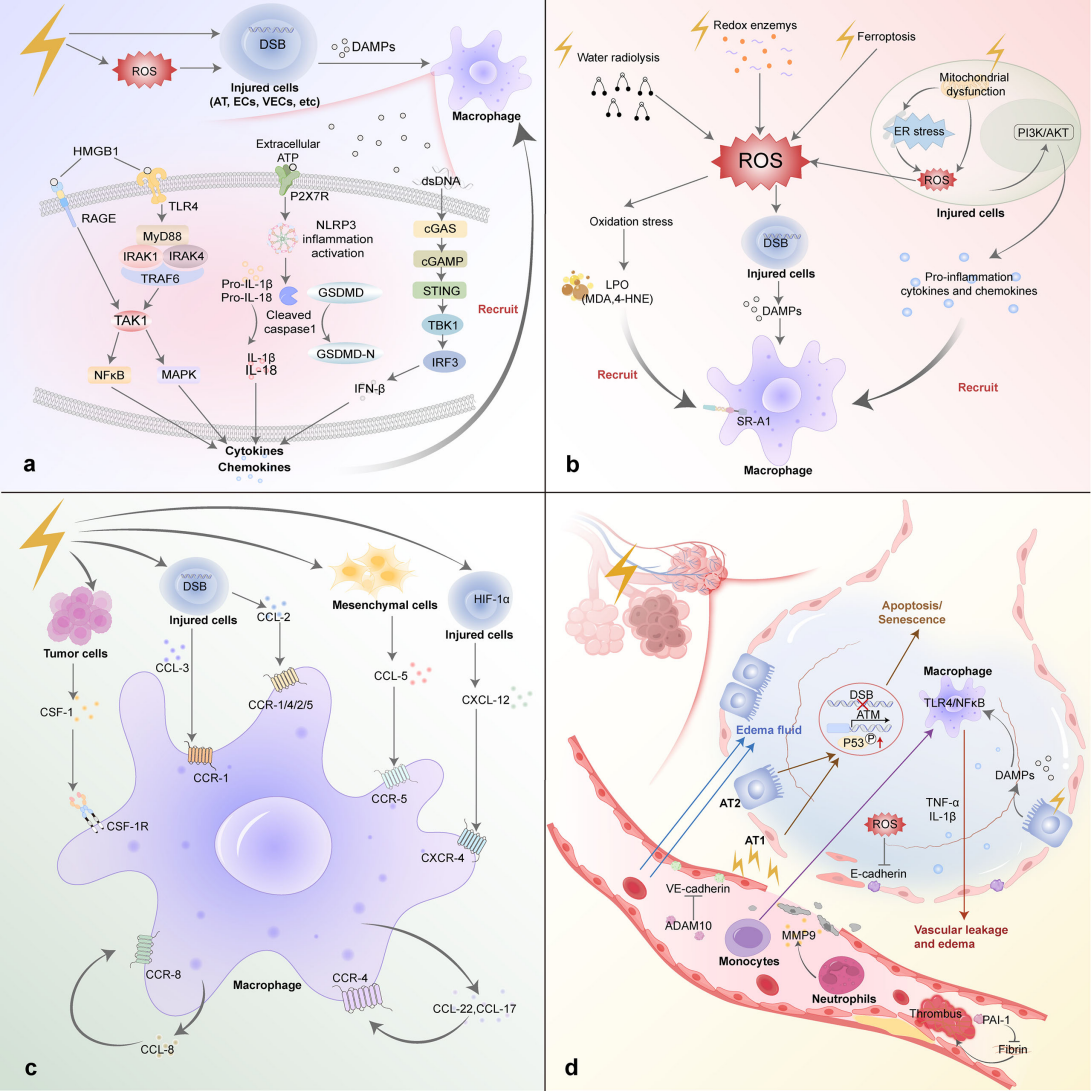
Mechanisms of macrophage recruitment in radiation-induced lung injury. **(A)** Radiation induces DSB and oxidative stress, triggering apoptosis, necroptosis, and ferroptosis in alveolar epithelial cells and vascular endothelial cells. Released DAMPs activate pattern recognition receptors (TLR4, RAGE, P2X7R, cGAS), stimulating multiple pathways to promote cytokine secretion and macrophage recruitment. **(B)** Radiation-generated ROS drive lipid peroxidation, producing reactive aldehydes (4-HNE, MDA) recognized by macrophage SR-A1 receptors. ROS also activate PI3K/Akt signaling pathway, collectively enhancing macrophage migration and infiltration. **(C)** Radiation upregulates chemokines (CSF-1, CCL2, CCL5, CXCL12, CCL3) from tumor, stromal, and injured cells, creating chemokine gradients that recruit and activate macrophages. Macrophage-derived CCL22/CCL17 and CCL8 establish positive feedback loops that amplify recruitment. **(D)** Radiation-induced DSB and ROS lead to epithelial cell apoptosis and tight junction disruption, compromising barrier integrity. Endothelial apoptosis and ADAM10-mediated VE-cadherin cleavage further increase leakage. DAMPs also upregulate pro-inflammatory cytokines, worsening leakage and edema. Neutrophils exacerbate barrier damage by releasing MMP-9, while radiation-induced PAI-1 upregulation inhibits fibrinolysis, promoting thrombosis and immune cell extravasation. ROS, reactive oxygen species; DSB, double-strand break; DAMP, damage-associated molecular pattern; ATI/II, alveolar type I/II epithelial cells; ECs, endothelial cells; VECs, vascular endothelial cells; HMGB1, high mobility group box 1; RAGE, receptor for advanced glycation end products; TLR, toll-like receptor; MyD88, myeloid differentiation primary response protein 88; IRAK, interleukin-1 receptor-activated kinase; TRAF, TNF receptor-associated factor; TAK, transforming growth factor-β-activated kinase; NF-κB, nuclear factor κ-light-chain-enhancer of activated B cells; MAPK, mitogen-activated protein kinase; GSDMD, gasdermin D; cGAS, cyclic GMP-AMP synthase; STING, Stimulator of interferon genes; TBK1, TANK-binding kinase 1; IRF, interferon regulatory factor; ER, endoplasmic reticulum; LPO, lipid peroxidation; MDA, malondialdehyde; 4-HNE, 4-hydroxynonenal; SR-A1, Scavenger receptor class A member 1; ATM, ataxia telangiectasia mutated; MMP, matrix metalloproteinase; PAI, plasminogen activator inhibitor.

The core mechanism by which RT induces RILI involves DNA double-strand breaks (DSBs) and indirect damage triggered by oxidative stress, leading to various forms of cell death, such as apoptosis, necrosis, and necroptosis, in alveolar epithelial cells (AECs) and vascular endothelial cells (VECs) (11). This process is accompanied by the release of a large number of damage-associated molecular patterns (DAMPs), including high mobility group box 1 (HMGB1), extracellular ATP, nDNA/mtDNA fragments, and calreticulin (12, 13). HMGB1 can bind to TLR4 on the surface of macrophages (14), activating IRAK1/4 via MyD88, thereby triggering the activation of the NF-κB and MAPK pathways to promote the secretion of pro-inflammatory cytokines and chemokines that recruit macrophages (15, 16). Alternatively, HMGB1 can bind to RAGE to activate the same pathways and upregulate the levels of adhesion molecules required for macrophage migration (16). Extracellular ATP can bind to the purinergic receptor P2X7 to trigger the assembly of the NLRP3 inflammasome, upregulate pro-inflammatory factors by cleaving caspase-1 to enhance macrophage infiltration (17). It can also interact with the P2Y2 receptor to induce directional migration of macrophages to the sites of dying or dead cells (18). The DNA fragments accumulated in the cytoplasm due to radiation damage activate the DNA sensor cGAS, which promotes the synthesis of cGAMP and activates STING. This, in turn, facilitates the expression of type I interferon and related chemokines through TBK1 and IRF3, ultimately enhancing the recruitment and infiltration of macrophages (13) (**Figure 1a**).

#### 2.1.2 ROS-dependent lipid peroxidation and pro-migration signaling

After RT, tissues generate a large amount of reactive oxygen species (ROS). In addition to directly damaging AECs and VECs, leading to the release of DAMPs to recruit macrophages, ROS can also recruit macrophages through other mechanisms. Excessive production of ROS can lead to oxidative stress in lung tissue, subsequently triggering lipid peroxidation (19, 20). The resulting reactive aldehydes, such as 4-HNE and MDA (21, 22), can serve as oxidation-specific epitopes recognized by the macrophage scavenger receptor SR-A1 (23). This interaction promotes macrophage activation, proliferation, and infiltration into irradiated tissues. Additionally, studies have reported that the generation of intracellular ROS and sustained oxidative stress can activate the PI3K/Akt pathway (24). Pro-inflammatory cytokines mediated by PI3K signaling are involved in the migration and adhesion processes of macrophages, and these processes are crucial for the recruitment and infiltration of macrophages into inflammatory regions (25, 26) (**Figure 1b**).

#### 2.1.3 Chemokine secretion drives macrophage recruitment and activation, triggering an inflammatory cascade amplification

RT can upregulate multiple chemokines and mediate specific mechanisms to increase macrophage infiltration. RT promotes the secretion of CSF-1 by tumor cells (27), which binds to CSF-1R on the surface of macrophages, triggering receptor dimerization and autophosphorylation, activating downstream signaling cascades to regulate macrophage migration, proliferation, and survival (28). Research has found that RT can upregulate CCL2 by activating the cGAS-STING pathway in mouse lung tissue (10, 29). CCL2 can induce massive infiltration of macrophages by binding to CCR2 on their surface (it can also bind to CCR1, CCR4, and CCR5) (30, 31). Zheng et al. discovered that RT induces stromal cells to upregulate CCL5 via the STING pathway, promoting the recruitment of CCR5+ macrophages (32). In addition, RT can also upregulate SDF-1 (CXCL12) by inducing hypoxia (33), which binds to CXCR4 to achieve macrophage recruitment. Moreover, *in vivo* thoracic RT induces an increase in CCL3 in mouse lung tissues (34), and CCL3 attracts macrophages to the irradiated site by binding to CCR1 on their surface. These signaling axes collectively form a powerful chemokine gradient that not only recruits macrophages but also functionally “licenses” them *in situ* (**Figure 1c**).

#### 2.1.4 Macrophage-derived chemokines maintain a positive feedback loop

RT can also promote the recruited macrophages to release chemokines, thereby recruiting more macrophages. In irradiated lungs, CCL22/CCL17 produced by alveolar macrophages (AMs) binds to CCR4, attracting more monocytes and predisposing them towards a profibrotic program (35). After low-dose RT, a unique macrophage subset with high CCL8 expression emerges and promotes secondary macrophage infiltration (36). Additionally, IL-8 secreted by macrophages can also induce the production of CSF-1, forming a positive feedback loop that further promotes macrophage recruitment (37) (**Figure 1c**).

#### 2.1.5 Radiation leads to increased vascular permeability and immune cell extravasation

RT directly leads to damage of the alveolar-capillary barrier. In the epithelium, RT induces DSBs, activates ATM, and phosphorylates p53 (38, 39), ultimately leading to apoptosis/senescence of type I alveolar epithelial cell (AT I) and compensatory proliferation and abnormal differentiation of AT II (40, 41). Meanwhile, RT induces the production of a large amount of ROS, which downregulates epithelial tight junction proteins (e.g., E-cadherin) through oxidative stress, thereby disrupting barrier integrity (42, 43). At the basement membrane, RT leads to its fragmentation, sub-basement membrane edema, and cell shedding, resulting in the loss of structural support for epithelium and endothelium (11). In the endothelium, RT directly induces endothelial cell apoptosis, leading to their detachment, and activates the ADAM10 protease, which cleaves VE-cadherin in a dose-dependent manner, disrupting endothelial adhesion junctions and thereby increasing macromolecular leakage (44). In addition, secondary inflammatory cascades further exacerbate leakage. RT-induced release of DAMPs induces the expression of TNF-α and IL-1β through the TLR4/NF-κB pathway, thereby weakening endothelial tight junctions and aggravating medium vessel leakage and edema (45). Infiltrating neutrophils degranulate to release MMP-9, which directly degrades type IV collagen in the basement membrane and disrupts the physical support between epithelium and endothelium (46, 47). And RT-induced upregulation of PAI-1 expression inhibits fibrinolysis and promotes microthrombus formation, thereby worsening perfusion and facilitating additional immune cell extravasation (48) (**Figure 1d**).

### 2.2 Radiotherapy regulates time-dependent macrophage phenotypic polarization

Macrophages display substantial plasticity throughout the course of RILI, exhibiting stage-dependent and non-binary phenotypes that dynamically shift with disease progression and are essential for maintaining tissue homeostasis. In the early stage after RT, the resident macrophages in the lungs and monocyte-derived macrophages can be abnormally activated and polarized towards the M1 phenotype. With persistent stress, these cells differentiate into M2 macrophages. The mechanisms by which radiation drives macrophage phenotypic polarization are diverse and have been extensively validated. Based on current research, we have summarized the following key pathways:

#### 2.2.1 Direct radiation injury promotes macrophage polarization towards the M1 phenotype

**Figure 2:**
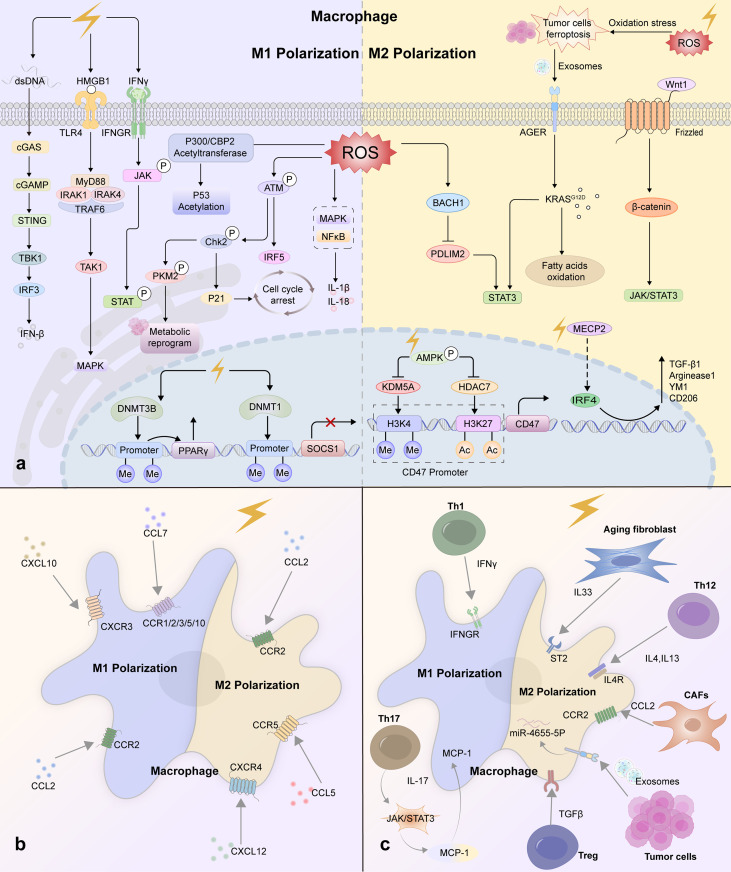
Mechanisms of macrophage polarization in radiation-induced lung injury. **(A)** Radiation induces early M1 polarization via dsDNA-cGAS-STING, HMGB1-TLR4, IFN-γ/JAK-STAT1 and ROS-ATM-Chk2/IRF5 pathways, plus p300/CBP acetyltransferase-mediated p53 acetylation signaling pathway. Late-stage M2 repolarization is driven by persistent ROS inhibiting PDLIM2 via BACH1 to activate STAT3 and exosome from ferroptotic tumor cells that macrophages internalize via AGER to activate STAT3 and fatty acid oxidation. Epigenetically, DNMT3B induced-methylation of PPARγ promoter and DNMT1 induced-methylation of SOCS1 promoter induce macrophage M1 polarization, while AMPK-CD47, MeCP2-IRF4, and Wnt/β-catenin-STAT3 pathways favor macrophage M2 polarization. **(B)** In early stage of RILI, radiation-induced CCL7, CXCL10, and CCL2 promote macrophage M1 polarization through CCR1/2/3/5/10, CXCR3, and CCR2, respectively. In late stage of RILI, radiation-induced CCL2, CCL5, and CXCL12 induce macrophage M2 repolarization via CCR2, CCR5, and CXCR4, respectively. **(C)** Th1-derived IFN-γ and Th17-derived IL-17 promote macrophage M1 polarization. Conversely, Th2-derived IL-4/IL-13, Treg-derived TGF-β, CAF-derived CCL2, senescent fibroblast-derived IL-33, and tumor cell-released exosome miR-4655-5p promote macrophage M2 polarization. IFNGR, interferon gamma receptor; Chk2, checkpoint kinase 2; PKM2, pyruvate kinase M2; DNMT, DNA methyltransferase; PPAR, peroxisome proliferator-activated receptor; SOCS1, suppressor of cytokine signaling 1; BACH1, BTB domain and CNC homolog 1; PDLIM2, PDZ and LIM domain-containing protein 2; AGER, advanced glycosylation end-product-specific receptor; KDM5A, lysine demethylase 5A; HDAC7, histone deacetylase 7; MECP, methyl-CpG-binding protein; CAFs, cancer-associated fibroblasts; MCP, monocyte chemoattractant protein.

Radiation-induced cellular necrosis and activation of the immune sensing system initiate and sustain multiple classical and non-classical inflammatory pathways, collectively promoting macrophage M1 polarization. Firstly, RT directly induces DSB, and the dsDNA released into the cytoplasm can be recognized by cGAS, thereby activating the cGAS-STING pathway. This drives the TBK1-IRF3 axis to mediate the expression of type I interferons (e.g., IFN-β) and inflammatory factors, ultimately facilitating macrophage chemotaxis and activation into the M1 phenotype (10). Secondly, RT induces damaged or dying cells to release DAMPs, which upregulate the expression of chemokines through the HMGB1/TLR4/MyD88 pathway, activate the JNK signaling pathway, and induce the activation of NF-κB/AP-1 transcription factors, thereby promoting macrophage M1 polarization (49–51). In addition, Liu et al. demonstrated in a fractionated X-ray thoracic irradiation model that RT significantly elevated the levels of SAA in lung tissue. This protein can specifically bind to the FPR2 receptor on the macrophage membrane, activating macrophages through the FPR2-Rac1-NF-κB pathway and promoting their M1 polarization (52). Meanwhile, during the initial stage of RT, the local concentration of IFN-γ increases, which can further enhance the M1 polarization of macrophages through the JAK-STAT1 pathway (53) (**Figure 2a**).

#### 2.2.2 Biphasic effects of ROS: Inducing M1 polarization in the early stage and M2 repolarization in the late stage

Recent studies have confirmed that during the early stage after RT, a rapid burst of ROS drives macrophage M1 polarization. Li et al. demonstrated that ROS phosphorylate PKM2 via the ATM-Chk2 pathway and upregulate p21, thereby supporting macrophage M1 polarization by promoting glycolysis and cell cycle arrest (54). Similarly, NOX2-derived ROS activate ATM and stabilize IRF5, driving the pro-inflammatory M1 phenotype in macrophages (55). Ferroptosis plays a pivotal role in acute RILI (56), with high ROS induced by iron overload promoting macrophage M1 polarization by enhancing p300/CBP activity and facilitating p53 acetylation (57). Additionally, radiotherapy-induced oxidative stress upregulates SENP3 to stabilize HIF-1α and promote PKM2 expression, thereby facilitating M1 macrophage polarization and the production of pro-inflammatory cytokines (58) (**Figure 2a**).

However, the persistently existing ROS in the advanced stage after RT can promote the repolarization of macrophages towards the M2 phenotype. Li et al. discovered that the expression of the tumor suppressor PDLIM2 in AMs can be downregulated by the ROS-activated transcriptional repressor BACH1, leading to the constitutive activation of the transcription factor STAT3 and driving AMs M2 polarization (59). Also, NOX4-induced ROS recruit macrophages and induce their M2 polarization by upregulating the production of cytokines dependent on the ROS/PI3K/Akt signaling pathway (60). Additionally, oxidative stress can trigger ferroptosis in tumor cells, resulting in the release of KRAS^G12D protein via exosomes. After being taken up by macrophages through AGER, it activates STAT3 signaling and fatty acid oxidation (FAO), promoting their M2 polarization (61) (**Figure 2a**).

#### 2.2.3 Chemokine reprogramming drives the repolarization of macrophages from the M1 phenotype to the M2 phenotype

Early after RT, chemokines derived from tissues and tumors can induce macrophage M1 polarization. Irradiated epithelial cells secrete CCL7, which binds to CCR1/2/3 or CCR5/10 on the surface of macrophages, thereby recruiting macrophages while promoting their M1 polarization (62). Seo et al. found that ionizing radiation (IR) could selectively increase the production of CXCL10, which, by binding to CXCR3 on the surface of macrophages, drives macrophage M1 polarization through the CXCL10-CXCR3 axis (63, 64). Ni et al. demonstrated that radiation could also upregulate the level of CCL2, which promotes macrophage recruitment and M1 polarization (10) (**Figure 2b**).

In the late stage after RT, continuous exposure to the radiation microenvironment leads to immune-suppressive signals that promote macrophage M2 polarization. Chiang et al. discovered that CCL2 secreted by irradiated tumor cells can recruit precursors of tumor-associated macrophages (TAMs) and simultaneously induce the polarization of existing M1 macrophages toward the M2 state via the CCL2-CCR2 axis (65). Irradiated tumor cells also secrete high levels of CCL5, which, upon binding to CCR5 on the macrophage surface, induces M2 polarization by upregulating SOCS3 and inhibiting the STAT3 signaling pathway (66). Additionally, CXCL12 (SDF-1) is another key chemokine involved in macrophages M2 polarization after RT, with studies demonstrating that blocking the CXCL12-CXCR4 axis effectively prevents the infiltration and M2 polarization of macrophages (67) (**Figure 2b**).

#### 2.2.4 Epigenetic regulation drives macrophage phenotypic transformation

Early after IR, epigenetic modifications can promote macrophage M1 polarization. Wu et al. demonstrated that IR can induce the expression of DNA methyltransferase 3β (DNMT3B) (68), which activates the NF-κB pathway by methylating the PPARγ1 promoter, thereby inducing macrophage M1 polarization (69, 70). Cheng et al. found that IR can also mediate hypermethylation of the SOCS1 promoter through DNA methyltransferase 1 (DNMT1), leading to the loss of SOCS1 expression, which negatively regulates the activation of the JAK2/STAT3 pathway and further promotes the formation of the macrophage M1 phenotype (71) (**Figure 2a**).

However, after long-term exposure to IR, continuous irradiation promotes macrophage M2 polarization through unique epigenetic pathways. Sun et al. discovered that IR activates AMPK, which, on one hand, increases H3K4 methylation at the CD47 promoter by downregulating KDM5A, and on the other hand, increases H3K27 acetylation at the CD47 promoter by inhibiting HDAC7. These dual modifications upregulate CD47 to promote macrophage M2 polarization (72). Multiple studies have shown that IR can upregulate the methyl-binding protein (MeCP2) (73–75), which drives macrophage M2 polarization by enhancing IRF4 expression (76). In addition, IR induces increased β-catenin transcriptional activity to activate the Wnt/β-catenin pathway (77), which promotes macrophage M2 polarization by upregulating STAT3 and activating JAK/STAT signaling (78, 79) (**Figure 2a**).

#### 2.2.5 The interaction within the immune microenvironment induces macrophage reprogramming

Early after RT, helper T cells in the immune microenvironment dominate macrophage M1 polarization. Th1 cells secrete IFN-γ, which binds to IFNGR on the surface of macrophages and activates the JAK/STAT1 signaling pathway, promoting macrophage polarization towards the M1 type (80, 81). Wang et al. found that Th17 cells also increased in patients who developed RP after RT (82), and the IL-17 secreted by them could further promote macrophage M1 polarization through the JAK/STAT3 pathway in an MCP-1-dependent manner (83) (**Figure 2c**).

In the late stage after RT, multiple types of cells in the immune microenvironment synergistically secrete factors to induce macrophage M2 polarization. IL-4 and IL-13 secreted by Th2 cells can induce macrophage M2 polarization through STAT6 (Tyr641) phosphorylation (84). Treg cells secrete TGF-β and IL-10 while inhibiting Th17 cell responses, jointly promoting macrophage M2 polarization (85, 86). Cancer-associated fibroblasts after high-dose irradiation promote macrophage M2 polarization through the CCL2-CCR2 pathway (87). Radiation-induced senescent fibroblasts secrete IL-33, which binds to the ST2 receptor on the macrophage surface, driving their conversion to the M2 type (88). Additionally, studies have found that exosomal miR-4655-5p secreted by lung cancer cells after IR also promotes macrophage proliferation and M2 polarization by affecting chemokine activity and cytokine signaling pathways (89) (**Figure 2c**).

### 2.3 Radiotherapy regulates macrophage function through metabolic reprogramming

RT reshapes glucose, lipid, amino acid, and mitochondrial metabolism in the irradiation environment. Macrophages integrate these signals to stabilize their phenotype and effector functions.

#### 2.3.1 Radiotherapy affects macrophage phenotype by regulating glucose metabolism

**Figure 3:**
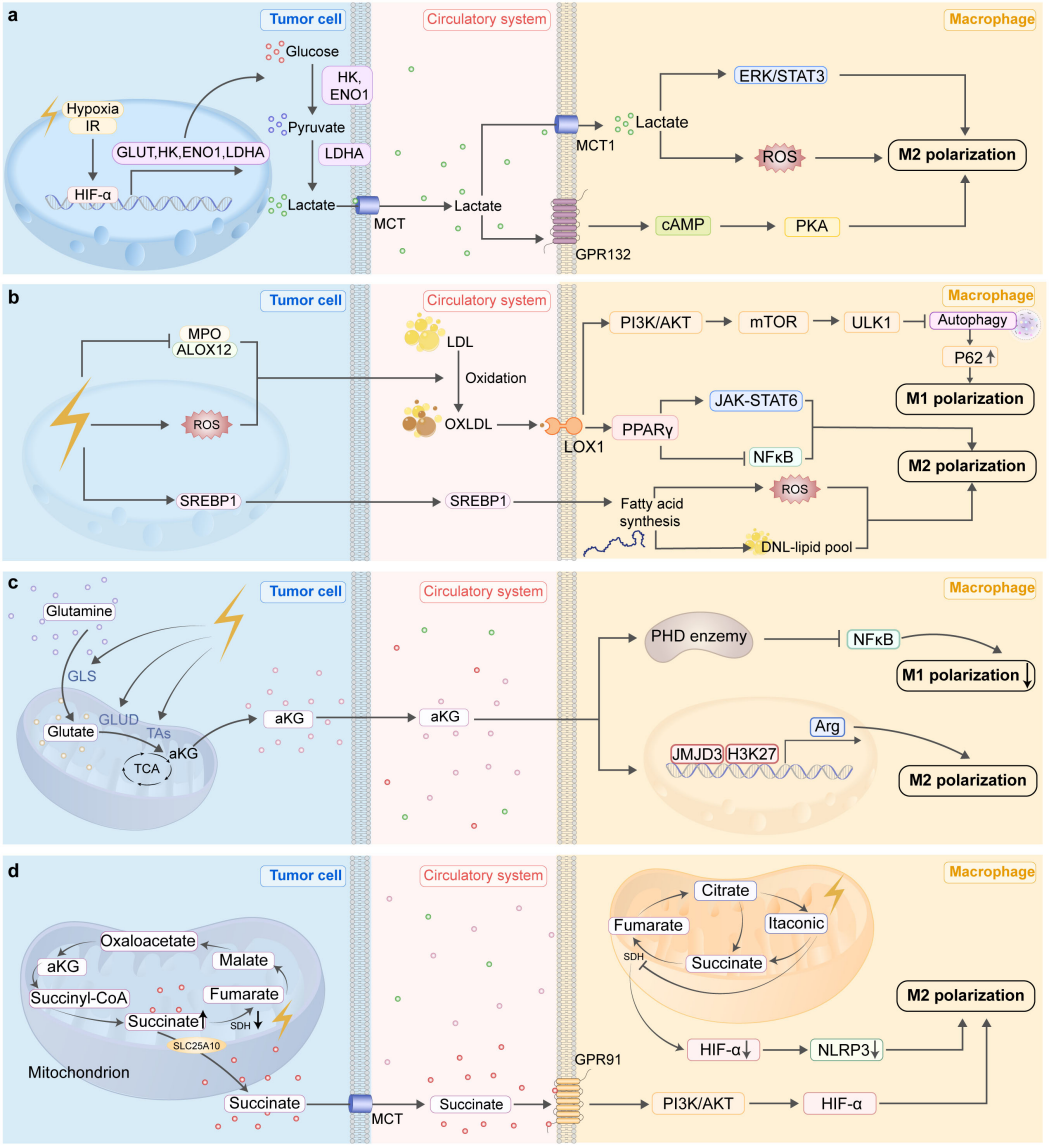
Radiation-induced metabolic reprogramming regulates macrophage polarization. **(A)** Radiation promotes the Warburg effect to increase lactate production of tumor cells. Lactate enters macrophages through MCT1, activating ERK/STAT3 signaling pathway and promoting ROS production, while also directly activating GPR132/cAMP/PKA signaling pathway, all promoting macrophage M2 polarization. **(B)** Radiation-generated oxLDL blocks macrophage autophagy by activating the PI3K/Akt/mTOR, leading to p62 protein accumulation and ultimately inducing macrophage M1 polarization. It also enhances the activity of PPARγ by inducing oxidative stress, promotes M2 polarization by activating JAK2/STAT6. Additionally, radiation-upregulated SREBP1 drives M2 polarization by inducing fatty acid synthesis and DNL. **(C)** Radiation upregulates glutamine metabolism in tumor cells. Glutamine-derived α-KG enhances fatty acid oxidation and JMJD3-mediated H3K27 demethylation to promote macrophage M2 polarization, while inhibiting NF-κB via PHD enzymes to restrict M1 polarization. **(D)** Radiation downregulates SDH expression in tumor cells, leading to succinate accumulation, which released into the extracellular space and binds to GPR91 on macrophages, activating PI3K and upregulating HIF-1α to promote M2 polarization. Itaconate inhibits SDH in macrophages, downregulates Hif-1α/NLRP3 to promotes macrophages M2 polarization. GLUT, glucose transporter; HK; hexokinase; ENO1, enolase 1; LDHA, lactate dehydrogenase A; MCT, monocarboxylate transporter; PKA, protein kinase A; MPO, myeloperoxidase; ALOX12, arachidonate 12-lipoxygenase; LDL, low-density lipoprotein; LOX, lysyl oxidase; mTOR, mechanistic target of rapamycin; ULK1, unc-51 like autophagy activating kinase 1; SREBP1, sterol regulatory element-binding protein 1; DNL, *de novo* lipogenesis; GLS, glutaminase; GLUD, glutamate dehydrogenase; TAs, transaminases; aKG, α-ketoglutarate; PHD, prolyl hydroxylase domain protein; JMJD3, jumonji domain-containing protein D3; Arg, arginine; SDH, succinate dehydrogenase.

RT can significantly alter the metabolic state of the local tissue microenvironment, with the most notable change being the enhancement of the Warburg effect. This effect elevates lactate levels in both tumor and surrounding normal tissues (50). After being transported into macrophages via MCT1, lactate can modulate the activity of various kinases and transcription factors, and directly promote M2 polarization by activating the ERK/STAT3 signaling pathway (90). Inhibition of this signaling pathway can reverse the lactate-induced M2 polarization effect (91). G protein-coupled receptor 132 (GPR132) is a specific receptor on the surface of macrophages for sensing lactate, which can specifically mediate lactate-induced M2 activation (92). Lactate released by cells can promote macrophage M2 polarization through the GPR132/cAMP/PKA pathway (93). In addition, lactate produced by tumor cells significantly promotes acidification of the tumor microenvironment (TME) (94), and the acidic environment can enhance the IL-4-driven phenotype in macrophages, thereby inducing their transformation into a tumor-promoting M2 phenotype (95). Another study has shown that lactate secreted by breast cancer cells can elevate intracellular ROS levels in macrophages via Nrf2-mediated mechanisms, ultimately inducing their M2 polarization (96) (**Figure 3a**).

#### 2.3.2 Radiotherapy disrupts lipid metabolism, thereby affecting macrophage polarization

The impact of RT on lipid metabolism is complex, and the resulting alterations in macrophage function are also intricate. For instance, literature has reported that standard radiation increases the production of oxLDL (97), which further activates the PI3K/AKT/mTOR pathway, blocking macrophage autophagy and leading to the accumulation of p62 protein; the accumulated p62 ultimately induces macrophage M1 polarization (98, 99).

On the other hand, standard radiation can enhance PPARγ activity through ROS-induced oxidative stress (97). PPARγ can bind to the promoters of pro-inflammatory genes and inhibit the activity of the transcription factor NF-κB to block M1 polarization (100). Also, it can promote IL-4-induced M2 polarization via the JAK2/STAT6 pathway (101). FLASH RT reduces PPARγ activation by eliminating the expression of lipid peroxidase, thereby inhibiting macrophage M2 polarization (97). In addition, RT can also significantly induce the expression and activity of the transcription factor sterol regulatory element-binding protein 1 (SREBP1) (102), which not only promotes the accumulation of free fatty acids (FFA) in the TME, driving macrophages towards an immunosuppressive and tumor-promoting M2 polarization (103), but also strengthens M2 polarization by inducing *de novo* lipogenesis (DNL), consuming NADPH, and elevating intracellular ROS (104) (**Figure 3b**).

#### 2.3.3 Radiotherapy regulates amino acid metabolism, thereby modulating macrophage polarization

RT can upregulate glutamine metabolism to meet cellular energy demands (105, 106). After entering cells via transport proteins, glutamine is converted into α-ketoglutarate (α-KG) by glutaminase (GLS), glutamate dehydrogenase (GLUD), and alanine/aspartate aminotransferases (TAs). On one hand, α-KG enhances FAO and oxidative phosphorylation (OXPHOS) capabilities, promoting macrophage M2 polarization through JMJD3-mediated demethylation of histone H3 lysine 27 (H3K27). On the other hand, it inhibits the activation of the NF-κB pathway in a prolyl hydroxylase domain (PHD) enzyme-dependent manner, thereby restricting macrophage M1 polarization (107, 108). In addition, glutathione (GSH) produced through glutamine metabolism can scavenge ROS, maintain the stability of the TME, and facilitate macrophage M2 polarization (109).

#### 2.3.4 Radiotherapy regulates macrophage polarization by inducing mitochondrial dysfunction to alter the tricarboxylic acid cycle

RT can regulate macrophage polarization by inducing cellular oxidative stress, damaging mitochondria, changing the mitochondrial TCA cycle and accumulating specific metabolites (110). Xiang et al. pointed out that IR can down-regulate succinate dehydrogenase (SDH) mediated by microRNA, leading to the accumulation of succinate (111). Extracellularly released succinate can specifically bind to the succinate receptor 1 (SUCNR1/GPR91) on the surface of macrophages, activating the PI3K pathway and significantly upregulating the expression of HIF-1α, ultimately promoting macrophage M2 polarization (112). In addition, RT can significantly increase the level of itaconate in the TME (113). An et al. found that itaconate can inhibit the activity of SDH, leading to downregulation of the Hif-1α/NLRP3 signaling pathway and promoting the polarization of AMs from the M1 phenotype to the M2 phenotype (114) (**Figure 3d**).

## 3 Adverse effects of macrophages on radiation pneumonitis

### 3.1 Acute inflammatory phase

#### 3.1.1 Initiation and amplification of inflammation

**Figure 4:**
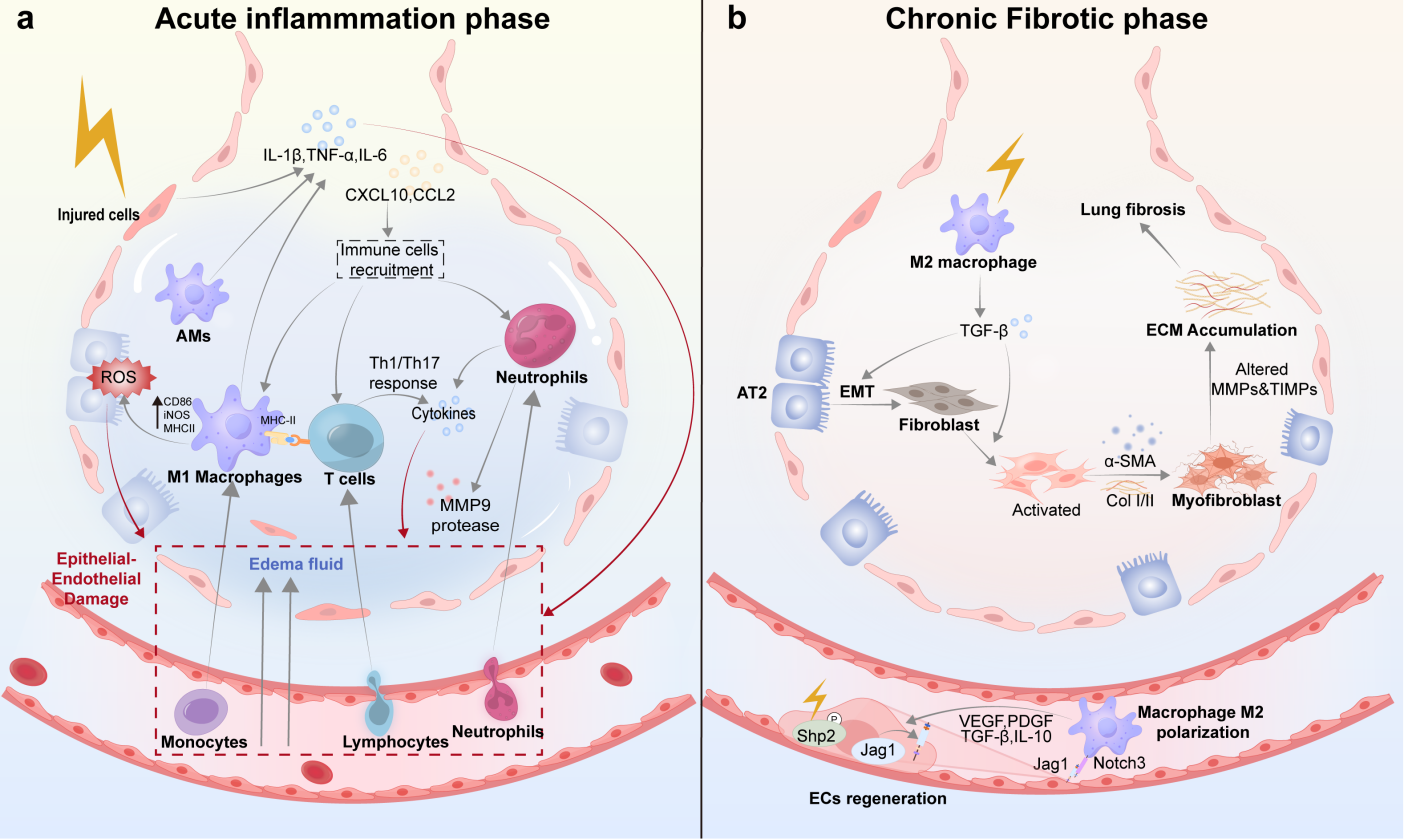
Roles of macrophages in different stages of radiation-induced lung injury. **(a)** In the acute inflammatory phase, radiation induces epithelial damage and activates AMs and monocyte-derived macrophages, leading to M1 polarization. These macrophages release ROS and pro-inflammatory cytokines (e.g., IL-1β, TNF-α, IL-6), as well as chemokines (e.g., CXCL10, CCL2). This not only amplifies the damage but also recruits immune cells. The cytokine storm, along with MMP-9 released by neutrophils, disrupts the epithelial-endothelial barrier, resulting in edema and exacerbating the inflammatory response. T cell activation further promotes this process through Th1/Th17 responses. **(b)** In the chronic fibrotic stage, macrophages polarize into the M2 phenotype, secreting TGF-β, VEGF, PDGF, and IL-10. They interact with ECs through the Jag1/Notch3 signaling pathway to support endothelial regeneration while maintaining pathological repair. TGF-β signaling induces EMT in type II alveolar epithelial cells and activates fibroblasts, which differentiate into myofibroblasts. Excessive deposition of type I/II collagen, coupled with an imbalance between MMPs and TIMPs, ultimately leads to excessive accumulation of ECM and irreversible pulmonary fibrosis. EMT, epithelial-mesenchymal transition; α-SMA, α-smooth muscle actin; Col I/II, collagen type I/II; TIMP, tissue inhibitor of metalloproteinases; ECM, extracellular matrix; Shp2, src homology region 2-containing protein tyrosine phosphatase 2; Jag1, jagged 1.

During the acute phase of RILI, macrophages play a central role in initiating and amplifying the inflammatory response. Radiation-induced DNA damage and oxidative stress can activate AMs and prompt peripheral monocytes to migrate to the lungs and differentiate into M1-type pro-inflammatory macrophages (2, 115). Activated M1-type macrophages highly express CD86, iNOS, and MHC-II, and release large amounts of pro-inflammatory cytokines such as IL-1β, TNF-α, and IL-6 (116), forming a “cytokine storm” that significantly exacerbates alveolar-capillary barrier disruption and local edema (117). Moreover, studies have confirmed that inhibiting the secretion of these pro-inflammatory factors through mechanisms such as selective depletion of pulmonary macrophages can reduce alveolar permeability, alleviate pulmonary edema, and mitigate tissue structural damage (118–120). Meanwhile, macrophages produce a large amount of ROS under conditions of radiation and hypoxia. On one hand, this further induces the irradiated tissues to release IL-1, IL-4, IL-6, TNF-α, and other cytokines involved in the occurrence of RP (121). On the other hand, the local accumulation of ROS causes VECs damage, dysfunction, and junction disruption, allowing inflammatory cells to traverse the endothelial barrier, exacerbating tissue inflammation, and worsening alveolar barrier destruction (122) (**Figure 4a**).

#### 3.1.2 Positive feedback driven by inflammatory factors

Previous studies have demonstrated that IR can selectively induce the production of CXCL10 (123) in mouse macrophages and significantly increase the secretion of CCL2 by macrophages in the presence of tumor cells (124). After RT in lung cancer patients, elevated levels of CXCL10 in peripheral blood and lung tissue can enhance the infiltration of T cells into the lungs to activate cytotoxic immunity (125, 126). Radiation-upregulated CCL2 also mediates the recruitment of T cells, neutrophils, and monocytes (127, 128). The recruited immune cells can exacerbate lung injury by releasing effector molecules. MMP-9 and proteases released by neutrophils can degrade the alveolar basement membrane and surfactant, disrupt the alveolar-capillary barrier, leading to alveolar collapse and protein leakage into the alveolar space, thereby further aggravating pulmonary edema (46, 47, 129). Additionally, excessive accumulation of immune cells may trigger a cytokine storm, resulting in the massive release of inflammatory mediators and ROS, which further cause pulmonary parenchymal damage and worsen the inflammatory response in the lungs (130) (**Figure 4a**).

#### 3.1.3 Antigen presentation and autoimmunity

Radiation can lead to cell necrosis and DNA damage, releasing a large amount of self-antigens such as oxidized lipids, apoptotic cell debris, and mitochondrial components. These antigens can be taken up by pulmonary macrophages and presented to T cells. Relevant studies have confirmed that the antigen-presenting function of macrophages is significantly upregulated after RT (52, 131). Previous studies have demonstrated that AMs and monocyte-derived macrophages can participate in antigen recognition by highly expressing CD1b molecules or Mincle receptors, thereby inducing sustained Th1/Th17 responses to exacerbate chronic inflammation (132, 133). Based on this, it is speculated that in RILI, M1 macrophages are very likely to present self-antigens released from damaged tissues to T cells through MHC-II or CD1-related mechanisms, subsequently activating Th1/Th17 responses and triggering persistent immune inflammation and autoimmune damage. This also suggests that the antigen presentation and T cell costimulatory axis may serve as potential intervention targets (**Figure 4a**).

### 3.2 Chronic fibrotic phase

#### 3.2.1 Repair imbalance

During the chronic fibrotic phase of RILI, M2-type macrophages are activated and secrete growth factors such as VEGF and PDGF, as well as anti-inflammatory factors like TGF-β and IL-10. These factors play a crucial role in endothelial cell regeneration, thereby promoting the repair of damaged blood vessels and matrix reconstruction (134–136). Studies have shown that irradiated endothelial cells can secrete Jag1, which activates the Notch signaling pathway on the surface of macrophages, inducing their M2 polarization (137). This subsequently forms a bidirectional macrophage-endothelial cell interaction network. While this network maintains vascular integrity and promotes regeneration, its excessive activation can lead to pathological repair and exacerbate the fibrotic process (**Figure 4b**).

#### 3.2.2 Extracellular matrix accumulation

During the RILF process, M2 macrophages can secrete various profibrotic factors such as TGF-β, PDGF, and IL-13 (138). Among these, TGF-β is the core profibrotic factor, which can be activated by radiation-induced ROS and exert its effects through the TGF-β/Smad pathway (139, 140). On one hand, it activates the transformation of fibroblasts into myofibroblasts, upregulating the expression of myofibroblast markers α-smooth muscle actin (α-SMA) and type I and type III collagen (COL1/III), driving continuous collagen production. On the other hand, it increases the expression of tissue inhibitors of metalloproteinases (TIMPs), shifting the MMP/TIMP balance towards extracellular matrix (ECM) deposition (141–143). Studies have confirmed that in preclinical RILI models, inhibiting M2 macrophage polarization can effectively limit fibroblast activation and collagen accumulation, thereby reducing the degree of fibrosis (144) (**Figure 4b**).

#### 3.2.3 Epithelial-mesenchymal transition

During the chronic fibrotic phase of RILI, TGF-β1 secreted by M2 macrophages serves as a key factor inducing epithelial-mesenchymal transition (EMT) in AECs. TGF-β1 activates the canonical SMAD2/3 signaling pathway (145) and may synergize with pathways such as Wnt/β-catenin (41), prompting the transformation of AEC II into cells with fibroblast-like properties, which subsequently differentiate into myofibroblasts (146). These cells secrete a large amount of ECM, leading to the continuous progression of tissue fibrosis (147, 148). Additionally, TGF-β can also upregulate the expression of IGFBP7, further enhancing the EMT process through the ERK signaling pathway (149). Therefore, targeting the TGF-β/Smad signaling pathway to reverse EMT has emerged as a potential therapeutic strategy for delaying RILF (**Figure 4b**).

## 4 Therapeutic strategies targeting macrophages

### 4.1 Inhibition of recruitment

**Figure 5:**
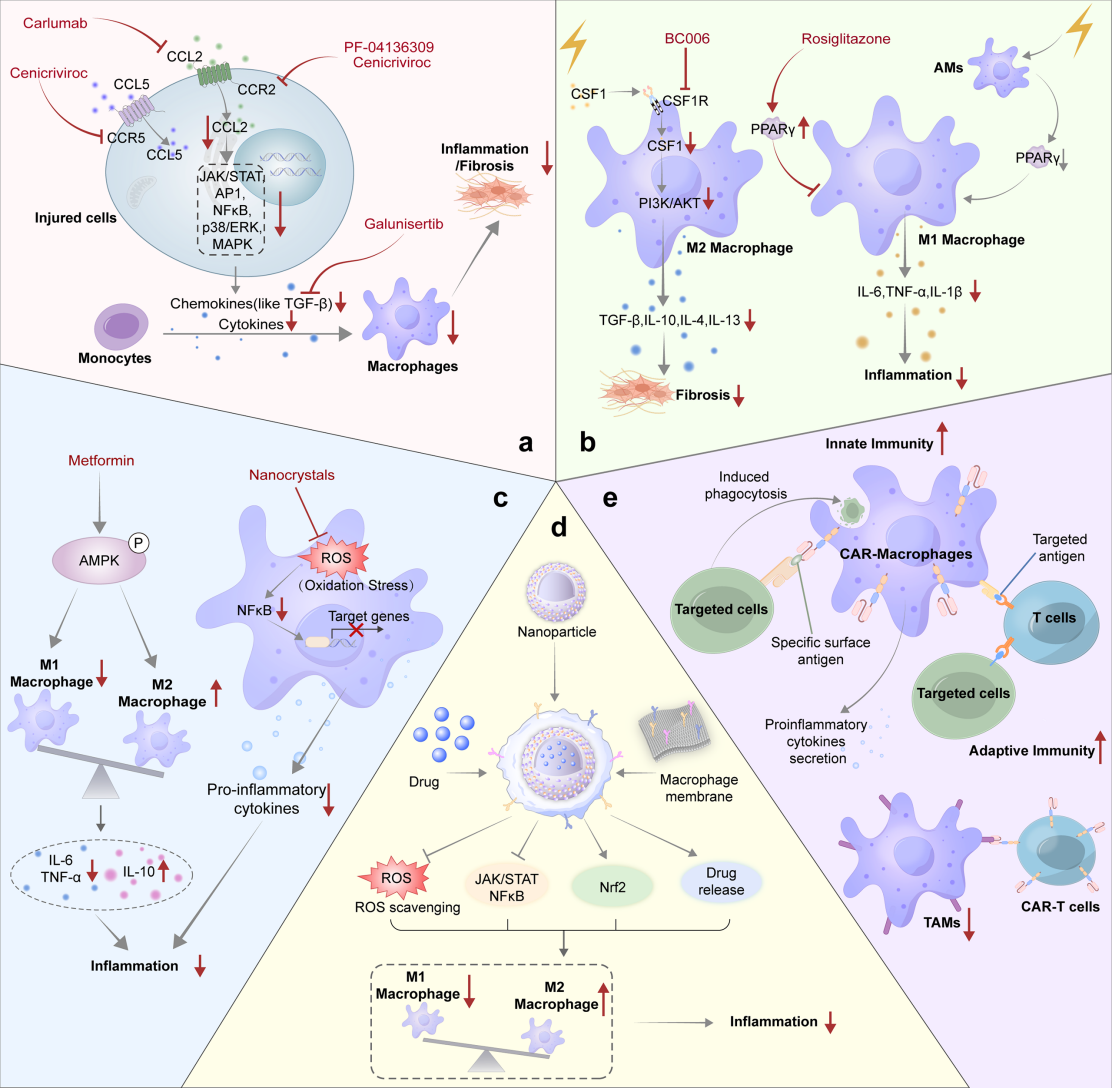
Therapeutic strategies targeting macrophages in RILI. **(a)** Inhibit recruitment: using Carlumab, Cenicriviroc, PF-04136309, Galunisertib and other drugs to block the CCL2/CCR2, CCL5/CCR5, and TGF-β pathways can prevent monocytes from migrating to lung tissue, reducing lung inflammation and fibrosis. **(b)** Phenotypic regulation: CSF-1R inhibitors such as BC006 inhibit M2 polarization, reducing lung fibrosis. PPARγ agonists such as rosiglitazone inhibit M1 polarization, reducing lung inflammation. **(c)** Metabolic intervention: Metformin activates AMPK, regulates macrophage polarization, and reduces the release of pro-inflammatory cytokines. Nanocrystals alleviate ROS-induced stress and downregulate the NF-κB signaling pathway, thereby reducing inflammation. **(d)** Delivery system: Macrophage membrane-coated nanoparticles enhance drug delivery to sites of inflammation, promote ROS scavenging, and regulate M1/M2 macrophage polarization. **(e)** Cell engineering: CAR-macrophages can recognize and target damaged lung areas and enhance phagocytosis through surface antigen guidance; CAR-T cells can eliminate tumor-associated macrophages, downregulate inflammatory pathways and reduce damage caused by macrophages. AMs, alveolar macrophages; AMPK, AMP-activated protein kinase.

Targeting the chemotactic process of monocytes-macrophages has become an important strategy for RILI treatment. CCL2 binds to CCR2, activating multiple signaling pathways, including JAK/STAT, AP-1, p38/ERK, MAPK, and NF-κB, thereby regulating the expression of a series of chemotaxis-and inflammation-related genes and promoting the migration of monocytes to lung tissue and their differentiation into macrophages. Preclinical studies have demonstrated that in a chronic obstructive pulmonary disease (COPD) model, blocking CCL2 can significantly improve lung function and alleviate alveolar structural destruction in mice (150). This effect provides insights for our future application of the CCR2 small-molecule antagonist PF-04136309 and the human anti-CCL2 monoclonal antibody Carlumab to inhibit monocyte recruitment to lung tissue for the treatment of pulmonary fibrosis. The CCL5-CCR5 signaling pathway has also been confirmed to play a crucial role in RILI and acute lung injury (ALI). In ALI models, CCR5 expression in lung tissue is significantly upregulated, and neutralizing CCL5 can markedly reduce leukocyte infiltration and exert lung-protective effects (151). Meanwhile, the dual CCR2/CCR5 antagonist Cenicriviroc significantly reduces monocyte/macrophage recruitment *in vivo* and markedly improves hepatic inflammation and fibrosis in nonalcoholic steatohepatitis NASH models, an effect that also provides insights for the treatment of pulmonary fibrosis (152, 153). In addition to targeting chemokine receptors, targeting cytokines also shows promising prospects. TGF-β, as a key pathological factor in RILI, its signal activation exacerbates RP and subsequent RILF. However, the application of the TGF-β receptor I kinase inhibitor Galunisertib (LY2157299) after thoracic RT can reduce collagen deposition and macrophage infiltration in lung tissue, significantly alleviating pulmonary inflammation and fibrosis (154) (**Figure 5a**).

### 4.2 Phenotypic regulation

Targeting macrophage phenotypic modulation represents a potential direction for intervening in RILI. The CSF-1/CSF-1R signaling pathway is crucial for macrophage survival and M2 polarization by activating the downstream PI3K/AKT pathway (28, 155). Preclinical studies have demonstrated that CSF-1R inhibitors (e.g., PLX3397 and BLZ945) can promote apoptosis of TAMs and reduce the expression of M2 markers like CD206 and IL-10, thereby attenuating the pro-fibrotic effects of M2-like macrophages to alleviate RILF (156, 157). The anti-CSF-1R monoclonal antibody BC006 can significantly reduce the number of inflammatory cells in the lungs, delay the progression of fibrosis, and improve pulmonary function indicators. Besides, as a key nuclear receptor transcription factor in macrophages, PPARγ’s agonist rosiglitazone can inhibit cigarette smoke-induced M1 polarization and reduce the intrapulmonary M1/M2 ratio in mouse models of COPD, thereby alleviating airway inflammation and lung function impairment (158). Although current research on PPARγ agonists and CSF-1R inhibitors in RILI models remains limited, both demonstrate promising application prospects in mitigating excessive early-stage inflammation and improving late-stage pulmonary tissue remodeling (**Figure 5b**).

### 4.3 Metabolic intervention

In terms of metabolic intervention, metformin, as a classic metabolic regulator, can remodel cellular energy metabolism by activating the AMPK signaling pathway and inhibiting key glycolytic enzymes (e.g., PFKFB3) (159). In ALI models, metformin has been found to reduce the levels of pro-inflammatory cytokines in macrophages, elevate the level of the anti-inflammatory cytokine IL-10, simultaneously downregulate the expression of the M1-type marker iNOS, and upregulate the expression of the M2-type marker Arg1, thereby alleviating pathological damage in lung tissue (160). In addition, given that IR can lead to oxidative damage, nanoparticles with antioxidant activity have emerged as a novel strategy. The CeO2/Mn3O4 composite nanocrystals constructed by Han et al. and the Co-Mn composite oxide nanoparticles prepared by Yang et al. can efficiently scavenge various ROS, inhibit the overactivated NF-κB inflammatory signaling pathway, and reduce the secretion of pro-inflammatory cytokines by pulmonary macrophages, thereby protecting lung tissue from radiation-induced oxidative damage (161, 162). Although metformin and nanotherapies are mostly studied in models of ALI or pulmonary fibrosis, their mechanisms of macrophage regulation provide important references for the prevention and treatment of RILI (**Figure 5c**).

### 4.4 Delivery systems

Leveraging the characteristics of macrophages in evading immune recognition and migrating toward inflammatory regions, wrapping nanoparticles with macrophage membranes can significantly enhance drug targeting (163). Zhao et al. utilized macrophage membrane-encapsulated mesoporous polydopamine nanoparticles to deliver peipeimine. The constructed MM@mPDA-PM NPs could target and accumulate in damaged lung tissues, promoting macrophage M2 polarization by downregulating the NF-κB and JAK/STAT pathways, thereby exerting anti-inflammatory effects (164). Zhao et al. utilized macrophage membrane-coated nanoparticles to deliver astaxanthin. Mϕ@Ast-nps could accumulate in damaged lung tissues, scavenge ROS, upregulate the Nrf2 antioxidant pathway, and inhibit the release of pro-inflammatory mediators (165). By leveraging the natural homing properties of M2 macrophage membranes to encapsulate ROS-responsive nanoparticles (NPs), a targeted delivery system to inflamed lung tissues was achieved. An inhalable biomimetic nanoplatform (M2M-SNPs) was developed, capable of scavenging ROS and neutralizing cytokines, thereby significantly ameliorating inflammatory infiltration and pulmonary edema (166). These studies provide insights into utilizing macrophage-related delivery systems to alleviate macrophage-mediated inflammation (**Figure 5d**).

### 4.5 Cell engineering

Cell engineering strategies directly modify macrophages themselves, enabling them to recognize specific targets and exert functions. Chimeric antigen receptor macrophage (CAR-M) therapy introduces specific recognition receptors into macrophages through genetic engineering, allowing them to target and act on specific cells (167). For example, Chen et al. introduced a CAR-like receptor targeting PD-L1 into macrophages, enabling them to effectively recognize and phagocytose PD-L1-positive target cells (168). This provides a new avenue for precision treatment of RILI—modifying macrophages to target radiation-damaged areas and regulating the local pulmonary immune microenvironment through “targeted clearance”. CAR-T therapy targeting macrophages also demonstrates significant potential. This strategy does not involve modifying macrophages themselves but rather engineering T cells to recognize and eliminate macrophages. For instance, Brian D. Brown designed CAR-T cells targeting F4/80, a specific surface marker on macrophages (F4.CAR-T), and discovered that F4.CAR-T could not only effectively clear TAMs but also reverse macrophage M2 polarization (169). In addition, engineered cellular carrier technology can utilize cells as “living robots” to precisely deliver therapeutic payloads (e.g., drugs, cytokines, and apoptotic signals) to the sites where macrophages are located. It has been reported that the use of enucleated mesenchymal stem cells (MSCs) with inherent homing properties as carriers for targeted delivery of plasmids encoding CAR to reprogram macrophages enables the *in vivo* preparation of CAR-M, promotes macrophage M1 polarization, and reshapes the intratumoral immune cell profile (170) (**Figure 5e**).

### 4.6 Future precision and predictive therapeutic strategies

Predicting and dynamically managing macrophage function represents the next frontier in RILI treatment. Because the therapeutic timing window is notoriously difficult to define (anti-inflammatory early vs. anti-fibrotic late), future strategies must rely on real-time predictive biomarkers. The integration of spatial transcriptomics and liquid biopsies assessing macrophage-derived exosomal profiles will likely enable clinicians to predict the exact polarization shift in a patient’s lungs in real time. In terms of recent therapeutic developments, beyond traditional metformin, novel precision drugs targeting specific metabolic pathways—such as inhibitors of oxidative phosphorylation (OXPHOS) and purine metabolism blockers—are showing immense promise in halting M2 repolarization. Additionally, the development of genetically engineered biomimetic nanovesicles and macrophage-membrane cloaked nanoparticles allows for the targeted co-delivery of anti-fibrotic drugs directly to the irradiated site, precisely dialing the macrophage functional state up or down as the disease phase dictates.

## 5 Challenges and future directions

RT occupies a pivotal and indispensable core position in the comprehensive treatment of lung cancer. However, its broader use has been accompanied by a rising incidence of RILI. Severe pneumonia, acute respiratory failure within RP and late pulmonary fibrosis can be lethal and may offset the benefits of RT. Macrophages, key effectors of innate immunity, play a dual and stage-dependent role in RILI, acting as both “destroyers” and “repairers”. Early after RT, irradiation recruits macrophages and promotes M1 polarization, exacerbating local inflammatory responses and exudation; later, macrophages shift toward M2 polarization through complex mechanisms, driving pulmonary fibrosis. Thus, an ideal approach would suppress M1 polarization early to limit inflammation while restraining M2 polarization later to prevent excessive fibrosis. At present, most of our therapeutic approaches targeting macrophages are still at the research stage, including regulating polarization direction, intervening in macrophage metabolism, applying cell engineering to modify macrophages, and utilizing nanoscale targeted delivery. However, their clinical breakthroughs remain limited.

We believe the main reasons are as follows. 1. The therapeutic timing window is hard to define. The course of RILI is prolonged, requiring diametrically opposite strategies at different stages, such as anti-inflammatory treatment in the early stage and anti-fibrotic treatment in the later stage. RILI evolves continuously, with no clear demarcation between acute inflammation and chronic fibrosis (171), and there are no reliable real-time biomarkers to indicate macrophage polarization status, which would assist doctors in determining the specific stage each patient is at and thereby selecting the most appropriate treatment regimen. Current diagnosis primarily relies on imaging and clinical symptoms, which is characterized by hysteresis (172). 2. Spatially specific targeting of lung macrophages is difficult. Systemic drug administration can affect macrophages in other tissues throughout the body, such as Kupffer cells in the liver and microglia in the central nervous system. This may lead to systemic immunosuppression, increase the risk of infection, or cause other unforeseen side effects. At present, although nanometer-targeted therapy has certain effects, accurately targeting macrophages in the lungs more precisely still faces identification challenges. This is primarily because macrophage markers are not absolutely specific and can change dynamically (173). Additionally, there is the issue of physiological barriers, as drugs need to traverse the pulmonary vascular endothelial barrier and be specifically and efficiently taken up by lung macrophages (174). 3. Patient heterogeneity is substantial: RT regimens, host factors, genetics, and baseline macrophage states vary widely (175). Currently, little is known about how to customize macrophage-targeted strategies based on the unique characteristics of each patient. 4. Macrophage modulation may conflict with tumor control. Systemic early-phase anti-inflammatory strategies that bias macrophages toward M2 could suppress anti-tumor immunity and raise recurrence risk. During the treatment process, it is necessary to repair normal lung tissue damage and maintain the killing effect on tumor cells, which requires the treatment strategy to possess extremely high spatial specificity, affecting only macrophages in the radiation-damaged area without influencing those in the TME.

Meanwhile, the combination of immune checkpoint inhibitors (ICIs) and RT has demonstrated significant synergistic anti-tumor effects in the treatment of lung cancer—ICIs can relieve the immunosuppressive state of the TME and enhance the immune recognition and elimination of tumor cells. Yet this regimen increases RILI risk, as heightened immunity can cause bystander damage to normal lung tissue and markedly raise pneumonitis rates (176, 177). A large-scale meta-analysis further revealed that the overall incidence of pneumonitis in RT combined with ICI therapy reached as high as 36% (177). Mechanistically, RT induces immunogenic tumor cell death and danger-signal release, promoting the aggregation of immune cells. On the other hand, PD-1/PD-L1 inhibitors (e.g., Pembrolizumab, Nivolumab, Atezolizumab) and other ICIs work by blocking the transmission of inhibitory signals in immune cells, thereby releasing the immune brake. Particularly, by targeting PD-L1 on the surface of macrophages, they relieve the inhibition of macrophage activation, prompting excessive activation of M1 macrophages and the production of a large amount of pro-inflammatory cytokines. Activated macrophages, serving as antigen-presenting cells, further stimulate T cells, while de-suppressed CD8+ T cells become highly cytotoxic. Together, they drive cytokine storm-like inflammation and tissue injury, worsening lung inflammation (177, 178). However, recent advances suggest that the relationship between immune checkpoint blockade and RILI is nuanced, and advanced delivery strategies may actually yield protective effects. For instance, recent research demonstrates that targeting mitochondrial energy metabolism can serve as a novel adjuvant. Zhou et al. developed mitochondria-targeted nanoparticles (e.g., IR-TAM@Alb and IR-LND@Lip) that effectively induce mitochondrial dysfunction, leading to the dual downregulation of PD-L1 and TGF-β. This approach not only sensitizes the tumor to radiotherapy and promotes a “hot” immune microenvironment but also actively inhibits radiation-induced pulmonary fibrosis by depressing collagen and TGF-β secretion (179, 180). Furthermore, the use of tumor microenvironment-responsive nanoplatforms can synergistically remodel cellular metabolism to overcome drug resistance without triggering the widespread autoimmune bystander effects typical of systemic ICIs (181). These targeted approaches represent a critical paradigm shift: ICIs can maximize anti-tumor immunity while mitigating the risk of RILI. Therefore, how to clinically balance the enhanced anti-tumor effects of ICI combined with RT against the risk of RILI it brings has become a critical scientific issue that urgently needs to be addressed (182). Although the combination of immune checkpoint blockade and macrophage reprogramming strategies can enhance the tumor control rate during thoracic RT combined with anti-PD-1 therapy, it is still associated with a relatively high risk of pneumonia (183, 184). The application of CSF-1R inhibitors (e.g., FF-10101) can reprogram macrophage subsets and increase intratumoral CD8^+^ T cells, thereby enhancing anti-tumor immune activity (185, 186). Furthermore, the bifunctional fusion protein M7824 (Bintrafusp alfa) targeting both PD-L1 and TGF-β, when combined with RT, has demonstrated the ability to reprogram the TME and overcome immune evasion in preclinical studies (187), and may also alleviate RILI. However, the clinical translation potential of these synergistic strategies still requires further research and validation.

In sum, macrophages are central to RILI pathogenesis and represent promising therapeutic targets. To prevent ICIs from exacerbating RILI, future research should place particular emphasis on the core role of macrophages in immunopathology, focusing on macrophage regulation to enhance the efficacy of cancer therapy while minimizing the risk of RILI and improving patients’ quality of life.

## Glossary

RILI: Radiation-induced Lung Injury
RT: Radiotherapy
RP: Radiation Pneumonitis
RILF: Radiation-induced Lung Fibrosis
AECs: Alveolar Epithelial Cells
VECs: Vascular Endothelial Cells
ATI: Alveolar Type I Epithelial Cells
ATII: Alveolar Type II Epithelial Cells
ROS: Reactive Oxygen Species
ICI: Immune Checkpoint Inhibitor
DAMPs: Damage-associated Molecular Patterns
DSB: DNA Double-strand Breaks
HMGB1: High Mobility Group Box 1
nDNA: Nuclear DNA
mtDNA: Mitochondrial DNA
TLR4: Toll-like Receptor 4
IRAK: Interleukin-1 Receptor-activated Kinase
MyD88: Myeloid Differentiation Primary Response Protein 88
RAGE: Receptor for Advanced Glycation End Products
STING: Stimulator of Interferon Genes
TBK1: Tank-binding Kinase 1
IRF3: Interferon RegulatoryFactor 3
4-HNE: 4-hydroxy-2-nonenal
MDA: Malondialdehyde
AMs: Alveolar Macrophages
ATM: Ataxia Telangiectasia Mutated Gene
ECM: Extracellular Matrix
SAA: Serum Amyloid A
FAO: Fatty Acid Oxidation
IR: Ionizing Radiation
TAMs: Tumor-associated Macrophages
DNMT3B: DNA methyltransferase 3β
PPARγ: Peroxisome Proliferators-activated Receptor γ
DNMT1: DNA methyltransferase 1
MeCP2: methyl-binding protein 2
SASP: Senescence-associated Secretory Phenotype
TIME: Tumor Immune Microenvironment
CAFs: Cancer-associated Fibroblasts
GPR: G Protein-coupled Receptor
TME: Tumor Microenvironment
oxLDL: Oxidized Low-density Lipoprotein
FFA: Free Fatty Acids
SREBP1: Sterol Regulatory Element-binding Protein 1
DNL: *De Novo* Lipogenesis*;* GLS, Glutaminase
GLUD: Glutamate Dehydrogenase
OXPHOS: Oxidative Phosphorylation
PHD: Prolyl Hydroxylase Domain
GSH: Glutathione
TCA cycle: Tricarboxylic Acid cycle
SDH: Succinate Dehydrogenase
SUCNR: Succinate Receptor
GPX4: Glutathione Peroxidase 4
α-SMA: α-Smooth Muscle Actin
TIMPs: Tissue Inhibitors of Metalloproteinases
EMT: Epithelial-mesenchymal Transition
COPD: Chronic Obstructive Pulmonary Disease
ALI: Acute Lung Injury
NPs: Nanoparticles
CAR-M: Chimeric Antigen Receptor Macrophage
CAR-T: Chimeric Antigen Receptor T-cell
MSCs: Mesenchymal Stem Cells.
